# Minimally important differences for the EORTC QLQ-C30 in prostate cancer clinical trials

**DOI:** 10.1186/s12885-021-08609-7

**Published:** 2021-10-07

**Authors:** Eva M. Gamper, Jammbe Z. Musoro, Corneel Coens, Jean-Jacques Stelmes, Claudette Falato, Mogens Groenvold, Galina Velikova, Kim Cocks, Hans-Henning Flechtner, Madeleine T. King, Andrew Bottomley, on behalf of the EORTC Genito-Urinary Tract Cancer Group and Quality of Life Groups

**Affiliations:** 1 Innsbruck Institute of Patient-centered Outcome Research (IIPCOR), Innsbruck, Austria; 2 European Organisation for Research and Treatment of Cancer (EORTC), Brussels, Belgium; 3 Department of Radiation Oncology, University Hospital Zurich, Zurich, Switzerland; 4 Department of Public Health, University of Copenhagen, and Bispebjerg Hospital, Copenhagen, Denmark; 5 Leeds Institute of Cancer and Pathology, University of Leeds, St James’s Hospital, Leeds, UK; 6 Adelphi Value, Bollington, Cheshire, UK; 7 Clinic for Child and Adolescent Psychiatry and Psychotherapy, University of Magdeburg, Magdeburg, Germany; 8 University of Sydney, Faculty of Science, School of Psychology, Sydney, NSW Australia

**Keywords:** Health-related quality of life, HRQOL, HRQL, Interpretation of scores, MIDs, Patient-reported outcomes, PROs, European Organisation for Research and Treatment of Cancer

## Abstract

**Background:**

The aim of the study was to estimate the minimally important difference (MID) for interpreting group-level change over time, both within a group and between groups, for the European Organisation for Research and Treatment of Cancer Quality of Life Questionnaire Core 30 (EORTC QLQ-C30) scores in patients with prostate cancer.

**Methods:**

We used data from two published EORTC trials. Clinical anchors were selected by strength of correlations with QLQ-C30 scales. In addition, clinicians’ input was obtained with regard to plausibility of the selected anchors. The mean change method was applied for interpreting change over time within a group of patients and linear regression models were fitted to estimate MIDs for between-group differences in change over time. Distribution-based estimates were also evaluated.

**Results:**

Two clinical anchors were eligible for MID estimation; performance status and the CTCAE diarrhoea domain. MIDs were developed for 7 scales (physical functioning, role functioning, social functioning, pain, fatigue, global quality of life, diarrhoea) and varied by scale and direction (improvement vs deterioration). Within-group MIDs ranged from 4 to 14 points for improvement and − 13 to − 5 points for deterioration and MIDs for between-group differences in change scores ranged from 3 to 13 for improvement and − 10 to − 5 for deterioration.

**Conclusions:**

Our findings aid the meaningful interpretation of changes on a set of EORTC QLQ-C30 scale scores over time, both within and between groups, and for performing more accurate sample size calculations for clinical trials in prostate cancer.

**Supplementary Information:**

The online version contains supplementary material available at 10.1186/s12885-021-08609-7.

## Background

While the importance of assessment of patient-reported outcomes (PROs) to measure health-related quality of life (HRQOL) in cancer clinical trials is no longer an issue of debate, difficulties in understanding the meaningfulness of resulting scores [1–3] remain a barrier for using them to their full potential. Statistical significance of observed differences and changes does not necessarily equate to clinical relevance nor does it reflect the importance of that difference or change for a patient. The concept of minimal important difference (MID) as “the smallest difference in score in the outcome of interest that informed patients or informed proxies perceive as important, either beneficial or harmful, and which would lead the patient or clinician to consider a change in the management” [4] is but one important component in the provision of an interpretation framework which allows putting PRO results into perspective. The MID of an instrument transforms the metric of the score into a clinical experience which not merely makes score changes actionable on a patient level, but may provide decision thresholds in testing the relative efficiency of treatments and inform the calculation of required sample sizes and numbers needed to treat (NNT) [4, 5].

There are different ways for determining MIDs, the division in anchor-based and distribution-based methods being an overall methodological classification. Anchor-based methods link PRO scores to external criteria of clinical relevant change, such as patient or clinical ratings, whereas distribution-based methods only consider the statistical distribution of the scores, e.g. defining an MID as a change larger than a pre-defined variation of measurement error [6].

Both methods have their strengths and their weaknesses. The anchor-based approach for instance strongly relies on the selection of appropriate anchors which may vary between conditions and settings. The distribution-based approach lacks a patient or clinical perspective and clinically relevant changes might be much more sample dependent.

As King et al. (2011) [7] highlight, there is no universal MID but rather a set of MIDs for instruments and scales, different conditions and clinical settings and, furthermore, distinction needs to be made between guidelines for group-level and individual-patient level interpretation of PRO scores. Therefore, it is recommended that MID selection should not rely solely on a rule of thumb, but must take into account the pre-specified research question at hand and knowledge of existing MIDs applicable to study specific instrument or scale.

Being the most frequently used HRQOL measure in cancer research [8] for the European Organisation for Research and Treatment of Cancer (EORTC) Quality of Life Questionnaire Core 30 (QLQ-C30) a number of MID estimates have been provided. These include both, anchor-based approaches using patient ratings [9] and clinical variables as anchors [10] as well as distribution-based methods [11] using data pooled across studies and cancer sites. Acknowledging that MIDs might differ across scales, direction of change (improvement vs deterioration) and cancer sites, an ongoing EORTC project aims at expanding the portfolio of QLQ-C30 MIDs by adding MIDs for each scale for different cancer sites [12]. Here we focus on prostate cancer, which currently accounts for 21% of cancers in men in the US [13]. Localized prostate cancer may be cured with surgery or radiation therapy, while in advanced disease hormonal, chemotherapeutic, and radionuclide therapies target the delay of progression and the palliation of symptoms. The two disease situations entail different conglomerates of symptoms and HRQOL issues associated with either disease or treatment or both. Problems with urinary function is most frequently observed in patients with prostatectomy, bowel problems have been linked to radiation therapy, and problems with sexual function have been associated with surgical procedures, hormonal therapy, as well as with the disease itself. The extent of psychological distress imposed by symptoms and the impact of the disease on general HRQOL and functioning aspects differ across patient groups [14]. Considered that there is a lack of clear consensus on optimal treatment strategy in many curative and palliative clinical situations with regard to survival [15, 16], HRQOL parameters are essential in future treatment studies and in clinical decision making. To support the use of HRQOL outcomes in prostate cancer research and to improve the interpretation of HRQOL scores in this population we here present the following QLQ-C30 MIDs for this patient group: (1) MIDs for within-group change in HRQOL scores over time and (2) MIDs for between-group differences in HRQOL change over time.

## Methods

### Data description

Data were derived retrospectively from two EORTC phase III trials in prostate cancer. Trial 1 (EORTC 22961) evaluated long term or short term androgen suppression combined with irradiation in locally advanced prostate cancer [17]. Trial 2 (EORTC 22991) compared the effectiveness of radiation therapy with or without bicalutamide and goserelin in treating patients who have localized prostate cancer [18]. Both trials collected HRQOL longitudinally using the EORTC QLQ-C30.

### The EORTC QLQ-C30

The EORTC QLQ-C30 consists of 30 questions that form 15 scales, 5 of which are functioning scales (physical, role, emotional, social, and cognitive), 9 are symptom scales (fatigue, nausea and vomiting, pain, dyspnoea, insomnia, appetite loss, constipation, diarrhoea and financial difficulties) and one is a global health status/QoL scale. Trial 1 used version 2 of the EORTC QLQ-C30, whereas trial 2 used version 3. The two versions differ only in the response categories of questions 1–5, coded as yes/no in version 2, whereas in version 3 responses are provided on a four-point Likert scale from ‘not at all’ to ‘very much’ for all questions with the exception of the global health status and quality of life which are rated from 1 ‘very poor’ to 7 ‘excellent’. Scoring was done according to the scoring manual [19], with the means of the raw scores for each scale transformed to fall between 0 and 100. For consistency in signs, all scales were scored such that 0 represents the worst possible score and 100, the best possible score. The financial impact scale was omitted from the analysis.

### Clinical anchor

For each EORTC QLQ-C30 scale we selected several anchor from clinical variables (e.g. WHO performance status (PS)) that were available from the data sets were selected. This was done using cross-sectional correlations (either polyserial or polychoric correlation to ensure acceptable correlation of ≥|0.3|) between the scales and the anchors [20]. It was aimed at using several anchors for each EORTC QLQ-C30 scale to provide some assurance about the plausibility of the estimated MIDs. Clinical input was provided by a panel of four prostate cancer / HRQOL experts to assure clinical plausibility of statistically selected anchors. Please refer to Musoro et al. [12] for details on the anchor selection methodology.

### Definition of clinical change groups

As described in earlier publications on the project [12, 20–23] the three clinical change groups (CCGs) defined by an expert panel were: *(i)* deterioration (worsened by 1 anchor category), *(ii)* stable (no change in anchor category) and *(iii)* improvement (improved by 1 anchor category). Patients changing by ≥2 points in anchor categories were considered to have changed more than just “minimally” and hence were excluded from MID estimation.

### Data analysis

Analysis have been described in more detail in previous publications [12, 20–23]. In overall two approaches to MID estimation have been applied, the anchor based and the distribution based approach.

For the anchor-based approach change scores for each scale and anchor pair were computed across all *pairwise* time points and MIDs for improvement and deterioration were estimated by calculating the mean HRQOL change score of patients classified as improved and deteriorated respectively (within-group MIDs). To estimate between-group MIDs (i.e. the differences in change over time between two groups of patients) linear regression models were fitted, one for each scale. Generalized estimating equations (GEE) was used to correct for the effect of patients contributing changes scores to several CCGs (and more than one to specific CCG) [24] Furthermore, we checked whether MIDs varied by trial in a regression model. To account for multiple testing (EORTC QLQ-C30 scales) statistical significance was set at 1%.

For the distribution-based approach 0.3 SD, 0.5 SD and standard error of measurement (SEM) were estimated at t1 (i.e. before or on the first day of treatment). As an effect size (ES) measure within CCGs the means of the HRQOL change scores were divided by the standard deviations (SD) of the HRQOL change scores over all time points. ES of 0.2 were considered small, 0.5 moderate and ≥ 0.8 large [25] and only anchor-based MIDS with mean changes with ES between 0.2 and 0.8 were considered appropriate for inclusion as MIDs.

## Results

A total of 1937 patients were enrolled in both trials. Patient characteristics at baseline are summarised in Table 1. The median follow-up time (in months) for HRQOL saw 36.2 (SD = 23.4) and 38.5 (SD = 34.6) for trials 1 and 2 respectively. An overview of patient inclusion in the various analysis steps is summarised in Fig. A.1.

**Table 1:** Demographic and clinical characteristics of patients at baseline

			Total (***N*** = 1937)
	Trial 1 (***N*** = 1118)	Trial 2 (***N*** = 819)	
	**N (%)**	**N (%)**	**N (%)**
**Age**			
Mean (SD)	68.68 (6.47)	69.39 (5.75)	68.98 (6.18)
Median	69	70	70
Q1-Q3	64–73	66–74	65–74
**N (%)**			
**Performance status**			
0	936 (83.7)	721 (88)	1657 (85.5)
1	156 (14)	96 (11.7)	252 (13)
2	17 (1.5)	2 (0.2)	19 (1)
Unknown	9 (0.8)	0 (0)	9 (0.5)
**Chronic disease**			
None	686 (61.4)	321 (39.2)	1007 (52)
Cardiovascular only	258 (23.1)	214 (26.1)	472 (24.4)
Other or multiple	165 (14.8)	284 (34.7)	449 (23.2)
Unknown	9 (0.8)	0 (0)	9 (0.5)
**Country**			
Netherlands	609 (54.5)	260 (31.7)	869 (44.9)
France	143 (12.8)	199 (24.3)	342 (17.7)
Belgium	98 (8.8)	23 (2.8)	121 (6.2)
United Kingdom	29 (2.6)	86 (10.5)	115 (5.9)
Spain	43 (3.8)	59 (7.2)	102 (5.3)
Israel	46 (4.1)	15 (1.8)	61 (3.1)
Cyprus	0 (0)	58 (7.1)	58 (3)
Italy	33 (3)	24 (2.9)	57 (2.9)
Switzerland	26 (2.3)	23 (2.8)	49 (2.5)
Ireland	0 (0)	38 (4.6)	38 (2)
Turkey	35 (3.1)	0 (0)	35 (1.8)
Russia	28 (2.5)	0 (0)	28 (1.4)
Malta	27 (2.4)	0 (0)	27 (1.4)
Germany	0 (0)	14 (1.7)	14 (0.7)
Czech Republic	0 (0)	7 (0.9)	7 (0.4)
Poland	0 (0)	7 (0.9)	7 (0.4)
Luxembourg	0 (0)	6 (0.7)	6 (0.3)
Bosnia and Herzegovina	1 (0.1)	0 (0)	1 (0.1)

Fourteen potential clinical anchors were initially evaluated for the EORTC QLQ-C30 scales. After retaining anchors with cross-sectional correlation ≤0.3, and seeking clinical input to confirm their clinical relevance, PS and CTCAE diarrhoea were retained. PS was scored between 0 (no symptoms of cancer) and 4 (bedbound) and CTCAE diarrhoea graded between 0 (no toxicity) to 4 (life-threatening). As shown in Table 2, a clinical anchor was found for 7 of the 14 scales considered, with cross-sectional correlations ranging from 0.3 to 0.55 in absolute value, and the correlations between their change scores ranging from 0.2 to 0.4.

**Table 2:** Cross-sectional correlations of the EORTC QLQ-C30 scales with anchors, and correlations between their change scores

		Absolute scores		Change scores	
Scale	Anchor	N (N_**o**_)	Correlation	N (N_**o**_)	Correlation
Physical Functioning	Performance status	1732 (8131)	−0.35	1445 (24420)	−0.3
Role Functioning	Performance status	1732 (8130)	− 0.5	1445 (24462)	− 0.3
Social Functioning	Performance status	1731 (8082)	−0.36	1445 (24204)	−0.2
Pain	Performance status	1732 (8136)	−0.33	1445 (24502)	−0.13
Fatigue	Performance status	1732 (8125)	−0.4	1445 (24436)	−0.2
Global Quality of Life	Performance status	1728 (8036)	−0.33	1445 (23929)	−0.2
Diarrhoea	CTCAE Diarrhoea	1587 (7108)	−0.45	1305 (20784)	−0.3

N = number of patients and N_o_ = number of observations.

*Abbreviations:* CTCAE, common terminology criteria for adverse events

According to the anchor change scores, the majority of patients remained stable over time compared to patients who either improved or deteriorated (Table A.1). Anchor-based MIDs that are derived from anchor CCGs with a clinically important ES (≥ 0.2 and < 0.8) are summarised in Table 3. The full results across all CCGs are presented in Table A.2. Anchor-based MIDs were determined for deterioration in 7 EORTC QLQ-C30 scales, and in 3 scales for improvement. The MID estimates varied by scale, direction of change (improvement versus deterioration), and were always in the expected direction, i.e. positive versus negative mean change scores within the improvement versus deterioration CCGs respectively. Within-group MIDs (from the mean-change method) ranged from 4 to 14 points for improvement and − 13 to − 5 points for deterioration, while MIDs for between-group change (from the linear regression) ranged from 3 to 13 for improvement and − 10 to − 5 for deterioration. The interaction effects between the binary anchor variable and the trial indicator showed no statistically significant differences for both improving and deteriorating scores (results not shown). This implies the estimated MIDs did not depend on the trial. In comparison to the distribution-based estimates presented in Table 3, apart from the diarrhoea scale, anchor-based MIDs for improvement were closer to 0.3 SD. For deterioration, anchor-based MIDs for diarrhoea, physical and role functioning scales were closer to 0.5 SD, while estimates for the remaining scales ranged between 0.3 SD and 0.5 SD. Distribution-based estimates for all 14 EORTC QLQ-C30 scales that were considered in this study are presented in Table A.3.

**Table 3:** Summary of anchor-based and distribution-based MIDs

	Anchor-based				Distribution-based*		
	MID for within-group change		MID for between-group difference in change				
**Scale**	**Improvement**	**Deterioration**	**Improvement**	**Deterioration**	**0.3 SD**	**0.5 SD**	**1 SEM**
PPhysical Functioning	No MID	−11	No MID	−7	4.8	7.9	4.8
Role FunctioningRF	4	−13	5	−10	5.6	9.3	7.9
Social FunctioningF	4	−5	3	−4	4.7	7.8	5.7
Fatigue	No MID	−9	No MID	−7	5.6	9.3	7.6
Pain	No MID	−6	No MID	−5	5.4	8.9	6.7
Global Quality of Life	No MID	−7	No MID	−6	5.5	9.2	7.8
Diarrhoea	14	−9	13	−9	4.5	7.5	8

The within-group MIDs are derived from the mean change method and the between-group MIDs from the linear regression

‘no MID’ is used where no MID estimate is available either due to the absence of a suitable anchor or effect size < 0.2

* The distribution-based estimated were computed at the time point for the start of treatment;

The symptom scores were reversed to follow the functioning scales’ interpretation; i.e. 0 represents the worst possible score and 100 the best possible score

*Abbreviations:* ES, effect size; CTCAE, common terminology criteria for adverse events; SD = standard deviation; SEM = standard error of measurement

## Discussion

Our analyses were part of an EORTC project [12] on MID development for the QLQ-C30 scales in various cancer entities and adds prostate-specific MIDs to the EORTC MID portfolio.

The main results of the study are anchor-based MIDs for deterioration for seven QLQ-C30 scales (physical functioning, role functioning, social functioning, pain, fatigue, global quality of life, diarrhoea), and for improvement for three QLQ-C30 scales (role functioning, social functioning, diarrhoea) both for within-group and between-group differences. MIDs varied by scale and direction (between 5 and 13 points for deterioration and 4 and 10 points for improvement), whereby the direction was always in accordance with the anchor change category (i.e. anchor scores indicating a low health status were associated with lower HRQOL scores). This compares well to MIDs already developed in this EORTC project for head and neck cancer [22], advanced breast cancer [21], malignant melanoma [20], colorectal [23] and ovarian [24] as well as to other similar research [26–28]. With two exceptions (global quality of life, diarrhoea), these MIDs were larger for deterioration compared to improvements. This aligns with existing findings even beyond the QLQ-C30 [17, 20, 21], suggesting that patients may have a higher sensitivity to favourable differences [26, 29, 30]. However this effect is not universal as other studies have reported no systematic differences in the magnitude of change between deteriorating and improving scores [15, 19, 22].

Overall, our MID estimates, with few exceptions, lie between 5 and 10 points, which corresponds to the thresholds suggested by Osoba et al. in 1998 [9] where patients’ reports on subjective change were used as clinical anchors. While these thresholds had been developed in breast and small-cell lung cancer patients, they have also been observed in various other cancer sites [21–23, 26–28, 31].

There seems to be a certain universality of an MID of 5–10 points on QLQ-C30 scales, but smaller and larger MIDs have been repeatedly found, especially for role functioning [20, 23] including the present study. This highlights that the scales and different sites are not to be tarred with the same brush.

### Limitations

There are though some limitations to be considered when interpreting the presented results.

Most importantly, after careful evaluation of 14 potential clinical anchors, only the CTCA diarrhoea scale and the WHO PS were suitable for MID estimation as the others showed low correlations with HRQOL scales. A reason may be found in certain insensitivity of these rating systems to HRQOL differences due to a low interrater reliability in toxicity identification with CTCAE [32] or somewhat wide WHO PS categories (e.g. between *0- fully active* and *1-able to carry out light work*). Ideally, multiple anchors including patient self-reports which might be able to shed some light on the issue of subjectively perceived change on different scales would be considered. Furthermore, it has to be noted that in the present statistical approach ordinal scales are treated as interval scales, disregarding the fact that a difference between “not at all” and “a little” might be different from the difference between “quite a bit” and “very much”. This is where item-response-theory based methods can provide valuable information in future research. Finally, only two trials could be included, none of which was covering metastasised disease. Hence, the application of the here developed anchor-based MIDs to a prostate cancer population with stage IV disease needs to be done with caution. A further limitation is that, based on the available data, no anchor-based MIDs for improvement could be developed for some scales. This needs to be covered by future research along with the investigation of additional anchors to further approach the concept of minimal change. Meanwhile, the presented distribution-based MIDs may provide some guidance.

It is a strength of the present study, though, that MIDs did not vary across the different data sources, i.e. a trial in locally advanced prostate cancer on the effect of androgen suppression and a trial on effectiveness of radiation therapy with or without bicalutamide and goserelin in localized prostate cancer, indicating a certain stability of the estimated values. Our results may therefore support sound hypothesis for HRQOL in clinical trials targeting similar patient groups.

## Conclusion

In general, it is acknowledged that MIDs are dynamic and that we should not be expecting one single MID for each scale of an instrument, nor should we expect them to be the same across different conditions. Therefore, the proper application of MIDs always includes the careful selection of the most appropriate estimate, considering the specific condition and decision context. Note that the current findings are part of a larger project that aims to develop an evidence-based MID catalogue that is more refined than the commonly used single value rule-of-thumb. We aim to further perform a comprehensive synthesis of MID estimates to identify plausible ranges based on patterns across multiple cancer sites, and to expand the estimation methodology beyond retrospective clinical anchors.

In conclusion, the MIDs presented here contribute to the meaningful interpretation of group-level changes (mostly deterioration) on a set of QLQ-C30 scales in prostate cancer patients undergoing treatment and may facilitate more accurate sample size estimation in trials with HRQOL endpoints. They may also be useful benchmarks in clinical practice where they can help the early detection of patients with relevant changes of health status. Further research is needed to confirm our findings and to extend the MID set for improvements, which may be important to detect relevant in early stage prostate cancer and survivors.

## Supplementary Information

**Additional file 1.**

**Additional file 2.**

## Data Availability

The data that support the findings of this study are available from the corresponding author upon reasonable request (See https://www.eortc.org/data-sharing/)**.**Code is available upon request.

## Abbreviations

EORTC: European Organisation for Research and Treatment of Cancer
PROs: patient-reported outcomes
QLQ-C30: Quality of Life Questionnaire Core 30
CCGs: clinical change groups
CTCAE: common terminology criteria for adverse events
ES: effect size
HRQOL: health-related quality of life
MID: minimal important difference
NNT: numbers needed to treat
PA: pain
PS: performance status
SD: standard deviation
SEM: standard error of measurement
