# Supplementary material for: Minimally important differences for the EORTC QLQ-C30 in prostate cancer clinical trials
Source: BMC Cancer. 2021 Oct 7;21:1083. doi: 10.1186/s12885-021-08609-7 (PMC8496068; doi:10.1186/s12885-021-08609-7)
Supplement: Supplementary file 1 — Additional file 1. [file 12885_2021_8609_MOESM1_ESM.pdf]

Minimally important differences for the EORTC QLQ-C30 in prostate cancer clinical trials

Eva M Gamper[1]*, Jammbe Z Musoro[2]*, Corneel Coens[2], Jean-Jacques Stelmes[3], Claudette Falato[2], Mogens Groenvold[4], Galina Velikova[5], Kim Cocks[6], Hans-Henning Flechtner[8], Madeleine T King[9], Andrew Bottomley[2] on behalf of the EORTC Genito-Urinary Tract Cancer Group and Quality of Life Groups

[1]Innsbruck Institute of Patient-centered Outcome Research (IIPCOR), Innsbruck, Austria, eva.gamper@iipcor.org, ORCID ID: 0000-0002-1700-4054

[2]European Organisation for Research and Treatment of Cancer (EORTC), Brussels, Belgium

[3]Department of Radiation Oncology, University Hospital Zurich

[4]Department of Public Health, University of Copenhagen, and Bispebjerg Hospital, Copenhagen, Denmark

[5]Leeds Institute of Cancer and Pathology, University of Leeds, St James's Hospital, Leeds, UK.

[6]Adelphi Values, Bollington, Cheshire, UK

[8]Clinic for Child and Adolescent Psychiatry and Psychotherapy, University of Magdeburg, Magdeburg, Germany

[9]University of Sydney, Faculty of Science, School of Psychology, Sydney, NSW, Australia

*Joint first authors

Corresponding Author:

Eva M Gamper

Innsbruck Institute of Patient-centered Outcome Research (IIPCOR)

6020 Innsbruck

Austria

eva.gamper@iipcor.org

Table A.1: Number of patients (number of observations) by change scores of suitable anchors

Anchor change score	CTCAE Diarrhoea	Performance status
-4	-	1 (1)
-3	-	-
-2	7 (13)	19 (46)
-1	90 (404)	363 (1556)
0	1289 (20502)	1399 (20504)
1	60 (249)	452 (2237)
2	7 (49)	46 (202)
3	-	6 (45)
4	-	1 (8)

Since a patient can have multiple assessments, that patient can contribute to multiple anchor change score category.

Abbreviations: CTCAE, common terminology criteria for adverse

Table A.2: Means change HRQOL scores (effect sizes) from the mean change method and linear regression

		Mean change method[1]			Linear regression[2]	
**Scale**	**Anchor**	**Improvement (ES)**	**Stable (ES)**	**Deterioration (ES)**	**Improvement**	**Deterioration**
PF	Performance status	0.43 (02) [a]	-25 (-0.11)	-11.26 (-0.62)	3.21 [a]	-7.29
	SD	18.44	154	19.66		
RF	Performance status	3.59 (0.20)	-1.12 (06)	-12.88 (-0.67)	5.15	-108
	SD	23.59	17.43	26.74		
SF	Performance status	3.58 (0.21)	0.29 (02)	-4.77 (-0.29)	3.41	-3.60
	SD	21.42	16.40	22.57		
FA	Performance status	3.41 (0.17) [a]	-0.24 (-01)	-8.98 (-0.45)	49 [a]	-6.85
	SD	210	16.66	236		
PA	Performance status	1.87 (0.11) [a]	04 (00)	-6.18 (-0.35)	28 [a]	-57
	SD	23.54	16.97	25.26		
QL	Performance status	35 (0.17) [a]	-1.11 (-06)	-7.40 (-0.42)	42 [a]	-5.65
	SD	205	16.31	189		
DI	CTCAE Diarrhoea	13.78 (0.79)	0.37 (02)	-9.35 (-0.54)	138	-98
	SD	273	17.30	28.54		

[1]The mean change method is useful for interpreting within-group change over time

[2]The linear regression is useful for interpreting between-group differences in change over time

[a] These estimated change scores were not considered to summarise the MID estimate because their ES were either <0.2

The symptom scores were reversed to follow the functioning scales' interpretation; i.e. 0 represents the worst possible score and 100 the best possible score

Abbreviations: PF = physical functioning; RF = role functioning; SF = social functioning; FA = fatigue; PA = pain; QL = global health status; ES, effect size; CTCAE, common terminology criteria for adverse events; SD = standard deviation within the anchor change groups

Table A.3 Distribution-based estimates

Scale	0.2 SD	0.3 SD	0.5 SD	1 SEM	No. of patients
PF	3.2	4.8	7.9	4.8	1282
RF	3.7	5.6	9.3	7.9	1282
SF	3.1	4.7	7.8	5.7	1277
CF	3.3	5	8.4	7.1	1282
EF	3.7	5.6	9.3	7	1279
QL	3.7	5.5	9.2	7.8	1272
FA	3.7	5.6	9.3	7.6	1278
PA	3.6	5.4	8.9	6.7	1283
NV	1.3	2	3.3	4	1283
AP	2.3	3.5	5.8	5.3	1282
DY	4.1	6.1	10.2	8.4	1278
CO	3.7	5.5	9.2	7.6	1276
DI	3	4.5	7.5	8	1276
SL	5	7.5	12.5	10.9	1281

The distribution-based estimated were computed at t1; the time point for the start of treatment;

Abbreviations: PF = physical functioning; RF = role functioning; CF = cognitive functioning; EF = emotional functioning; SF = social functioning; FA = fatigue; PA = pain; NV = nausea/vomiting; QL = global health status; DY = dyspnoea; AP = appetite loss; SL; sleep disturbance; CO = constipation; DI = diarrhoea; SD = standard deviation; SEM= standard error of measurement
