# Supplementary material for: Minimally important differences for the EORTC QLQ-C30 in prostate cancer clinical trials
Source: BMC Cancer. 2021 Oct 7;21:1083. doi: 10.1186/s12885-021-08609-7 (PMC8496068; doi:10.1186/s12885-021-08609-7)
Supplement: Supplementary file 2 — Additional file 2. [file 12885_2021_8609_MOESM2_ESM.pdf]

Minimally important differences for the EORTC QLQ-C30 in prostate cancer clinical trials

Eva M Gamper[1]*, Jammbe Z Musoro[2]*, Corneel Coens[2], Jean-Jacques Stelmes[3], Claudette Falato[2], Mogens Groenvold[4], Galina Velikova[5], Kim Cocks[6], Hans-Henning Flechtner[8], Madeleine T King[9], Andrew Bottomley[2] on behalf of the EORTC Genito-Urinary Tract Cancer Group and Quality of Life Groups

[1]Innsbruck Institute of Patient-centered Outcome Research (IIPCOR), Innsbruck, Austria, eva.gamper@iipcor.org, ORCID ID: 0000-0002-1700-4054

[2]European Organisation for Research and Treatment of Cancer (EORTC), Brussels, Belgium

[3]Department of Radiation Oncology, University Hospital Zurich

[4]Department of Public Health, University of Copenhagen, and Bispebjerg Hospital, Copenhagen, Denmark

[5]Leeds Institute of Cancer and Pathology, University of Leeds, St James's Hospital, Leeds, UK.

[6]Adelphi Values, Bollington, Cheshire, UK

[8]Clinic for Child and Adolescent Psychiatry and Psychotherapy, University of Magdeburg, Magdeburg, Germany

[9]University of Sydney, Faculty of Science, School of Psychology, Sydney, NSW, Australia

*Joint first authors

Corresponding Author:

Eva M Gamper

Innsbruck Institute of Patient-centered Outcome Research (IIPCOR)

6020 Innsbruck

Austria

eva.gamper@iipcor.org

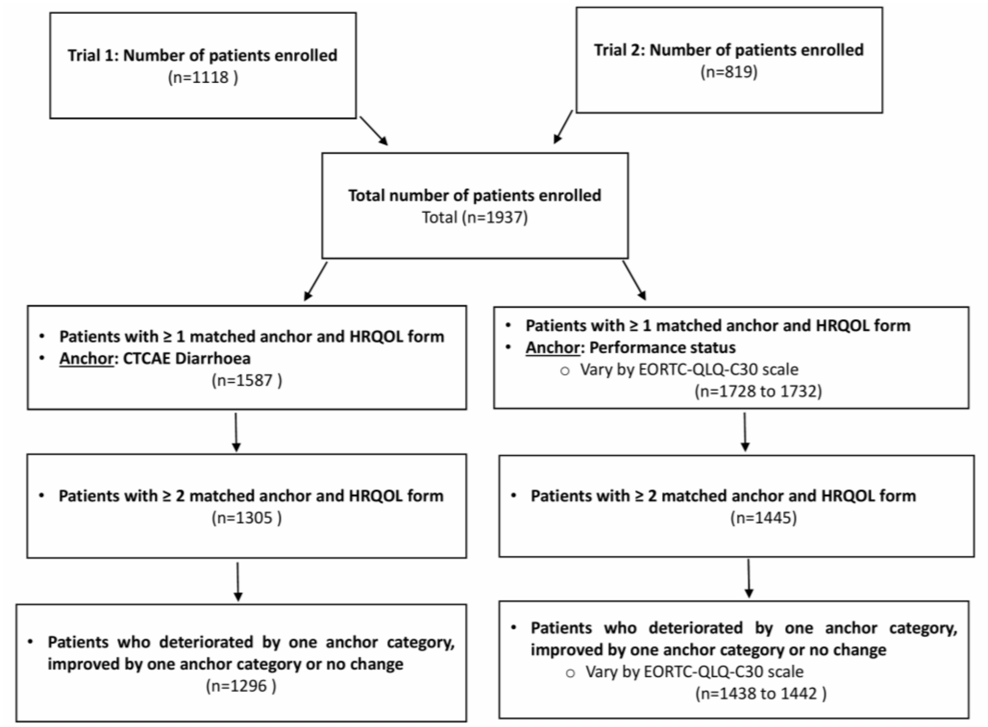

Figure A.1: An overview of patient inclusion.

Abbreviations: CTCAE; common terminology criteria for adverse events, HRQOL; health-related quality of life
